# Supplementary material for: Rapid assessment of psychological and epidemiological correlates of COVID-19 concern, financial strain, and health-related behavior change in a large online sample
Source: PLoS One. 2020 Nov 11;15(11):e0241990. doi: 10.1371/journal.pone.0241990 (PMC7657530; doi:10.1371/journal.pone.0241990)
Supplement: S1 File — (DOCX) [file pone.0241990.s001.docx]

Table of Contents

[Anxiety and Depressive Symptoms and Likely Diagnosis Compared to Historical Averages. 3](#_Toc50109325)

[Table S1. GAD-2 Scores Compared to Historical Normative Data 3](#_Toc50109326)

[Table S2. PHQ-2 Scores Compared to Historical Normative Data 3](#_Toc50109327)

[Mental Health and Epidemiological Predictors of COVID-19 Concern. 4](#_Toc50109328)

[Table S3: GAD-2 Predicting COVID-19 Concern. 4](#_Toc50109329)

[Table S4: PHQ-2 Predicting COVID-19 Concern. 5](#_Toc50109330)

[Table S5: Combined GAD-2 and PHQ-2 Model Predicting COVID-19 Concern. 6](#_Toc50109331)

[Table S6: Epidemiological Confirmed World Cases Predicting COVID-19 Concern. 7](#_Toc50109332)

[Table S7: Epidemiological Confirmed Country Cases Predicting COVID-19 Concern. 8](#_Toc50109333)

[Table S8: Combined Epidemiological Confirmed World and Country Cases Model Predicting COVID-19 Concern. 9](#_Toc50109334)

[Table S9: Combined Mental Health and Epidemiological Model Predicting COVID-19 Concern. 10](#_Toc50109335)

[Table S10: Loss of Employment Predicting COVID-19 Concern 11](#_Toc50109336)

[Table S11: Loss of Employment Predicting GAD-2 12](#_Toc50109337)

[Table S12: Loss of Employment Predicting PHQ-2 13](#_Toc50109338)

[Predictors of Degree of Adherence to Self-Quarantine Recommendations. 14](#_Toc50109339)

[Table S13: GAD-2 Predicting Degree of Self-Quarantine 14](#_Toc50109340)

[Table S14: PHQ-2 Predicting Degree of Self-Quarantine 15](#_Toc50109341)

[Table S15: Combined GAD-2 and PHQ-2 Model Predicting Degree of Self-Quarantine 16](#_Toc50109342)

[Table S16: COVID-19 Concern Predicting Degree of Self-Quarantine 17](#_Toc50109343)

[Table S17: Epidemiological Total World Cases Model Predicting Degree of Self-Quarantine 18](#_Toc50109344)

[Table S18: Epidemiological Total Country Cases Model Predicting Degree of Self-Quarantine 19](#_Toc50109345)

[Table S19: Epidemiological Total World and Country Cases Combined Model Predicting Degree of Self-Quarantine 20](#_Toc50109346)

[Table S20: Mental Health, COVID-19 Concern, and Epidemiological Confirmed World and Country Cases Combined Model Predicting Degree of Self-Quarantine 21](#_Toc50109347)

[Sample Descriptives 23](#_Toc50109348)

[Figure S1. Age Distribution 23](#_Toc50109349)

[Table S21. Participants by Country 24](#_Toc50109350)

# **Anxiety and Depressive Symptoms and Likely Diagnosis Compared to Historical Averages.**

## Table S1. GAD-2 Scores Compared to Historical Normative Data

| estimate | statistic | p-value | parameter | conf.low | conf.high | method | alternative |
| --- | --- | --- | --- | --- | --- | --- | --- |
| 3.3 | 57.34 | < .001 | 2073 | 3.22 | 3.39 | One Sample t-test | two.sided |

## Table S2. PHQ-2 Scores Compared to Historical Normative Data

| estimate | statistic | p-value | parameter | conf.low | conf.high | method | alternative |
| --- | --- | --- | --- | --- | --- | --- | --- |
| 2.6 | 41.93 | < .001 | 2073 | 2.52 | 2.67 | One Sample t-test | two.sided |

# **Mental Health and Epidemiological Predictors of COVID-19 Concern.**

## Table S3: GAD-2 Predicting COVID-19 Concern.

|  | **Unadjusted Model: COVID-19 Concern** | | | | **Adjusted Model: COVID-19 Concern** | | | |
| --- | --- | --- | --- | --- | --- | --- | --- | --- |
| *Predictors* | *Estimates* | *std. Error* | *Conf. Int (95%)* | *P-Value* | *Estimates* | *std. Error* | *Conf. Int (95%)* | *P-Value* |
| Intercept | 3.191 | 0.044 | 3.106 – 3.277 | **<0.001** | 282.502 | 46.141 | 192.067 – 372.937 | **<0.001** |
| GAD Score | 0.211 | 0.009 | 0.193 – 0.229 | **<0.001** | 0.219 | 0.009 | 0.201 – 0.236 | **<0.001** |
| Age |  |  |  |  | 0.014 | 0.002 | 0.011 – 0.018 | **<0.001** |
| Gender: Woman |  |  |  |  | 0.032 | 0.040 | -0.047 – 0.110 | 0.429 |
| Gender: Nonbinary |  |  |  |  | -0.022 | 0.111 | -0.240 – 0.195 | 0.840 |
| Gender: Prefer to Self Describe |  |  |  |  | 0.794 | 0.450 | -0.088 – 1.677 | 0.078 |
| Gender: Prefer not to Answer |  |  |  |  | -0.649 | 0.296 | -1.229 – -0.069 | **0.028** |
| Date |  |  |  |  | -0.015 | 0.003 | -0.020 – -0.010 | **<0.001** |
| **Random Effects** | | | | | | | | |
| σ^2^ | 0.66 | | | | 0.60 | | | |
| τ_00_ | 0.00 _country_region_ | | | | 0.00 _country_region_ | | | |
| N | 24 _country_region_ | | | | 24 _country_region_ | | | |
| Observations | 1990 | | | | 1990 | | | |
| AIC | 4831.208 | | | | 4663.369 | | | |

## Table S4: PHQ-2 Predicting COVID-19 Concern.

|  | **Unadjusted Model: COVID-19 Concern** | | | | **Adjusted Model: COVID-19 Concern** | | | |  |
| --- | --- | --- | --- | --- | --- | --- | --- | --- | --- |
| *Predictors* | *Estimates* | *std. Error* | *Conf. Int (95%)* | *P-Value* | | *Estimates* | *std. Error* | *Conf. Int (95%)* | *P-Value* |
| Intercept | 3.445 | 0.065 | 3.318 – 3.572 | **<0.001** | | 391.396 | 50.476 | 292.465 – 490.327 | **<0.001** |
| 'PHQ Score | 0.126 | 0.011 | 0.104 – 0.147 | **<0.001** | | 0.154 | 0.011 | 0.133 – 0.175 | **<0.001** |
| Age |  |  |  |  | | 0.014 | 0.002 | 0.010 – 0.017 | **<0.001** |
| Gender: Woman |  |  |  |  | | 0.109 | 0.043 | 0.025 – 0.194 | **0.011** |
| Gender: Nonbinary |  |  |  |  | | 0.151 | 0.119 | -0.083 – 0.385 | 0.207 |
| Gender: Prefer to Self Describe |  |  |  |  | | 0.747 | 0.487 | -0.207 – 1.701 | 0.125 |
| Gender: Prefer not to Answer |  |  |  |  | | -0.722 | 0.321 | -1.351 – -0.094 | **0.024** |
| Date |  |  |  |  | | -0.021 | 0.003 | -0.027 – -0.016 | **<0.001** |
| **Random Effects** | | | | | | | | | |
| σ^2^ | 0.78 | | | | | 0.71 | | | |
| τ_00_ | 0.02 _country_region_ | | | | | 0.01 _country_region_ | | | |
| N | 24 _country_region_ | | | | | 24 _country_region_ | | | |
| Observations | 1989 | | | | | 1989 | | | |
| AIC | 5156.801 | | | | | 4978.370 | | | |

## Table S5: Combined GAD-2 and PHQ-2 Model Predicting COVID-19 Concern.

|  | **Mental Health Model** | | | |
| --- | --- | --- | --- | --- |
| *Predictors* | *Estimates* | *std. Error* | *Conf. Int (95%)* | *P-Value* |
| Intercept | 292.506 | 47.792 | 198.834 – 386.177 | **<0.001** |
| GAD Score | 0.211 | 0.012 | 0.188 – 0.233 | **<0.001** |
| PHQ Score | 0.014 | 0.013 | -0.011 – 0.039 | 0.283 |
| Age | 0.015 | 0.002 | 0.011 – 0.018 | **<0.001** |
| Gender: Woman | 0.041 | 0.041 | -0.039 – 0.121 | 0.319 |
| Gender: Nonbinary | -0.030 | 0.116 | -0.257 – 0.196 | 0.793 |
| Gender: Prefer to Self Describe | 0.796 | 0.453 | -0.091 – 1.684 | 0.079 |
| Gender: Prefer not to Answer | -0.652 | 0.298 | -1.236 – -0.068 | **0.029** |
| Date | -0.016 | 0.003 | -0.021 – -0.011 | **<0.001** |
| **Random Effects** | | | | |
| σ^2^ | 0.61 | | | |
| τ_00_ _country_region_ | 0.00 | | | |
| N _country_region_ | 21 | | | |
| Observations | 1929 | | | |
| AIC | 4544.986 | | | |

## Table S6: Epidemiological Confirmed World Cases Predicting COVID-19 Concern.

|  | **Unadjusted Model: COVID-19 Concern** | | | | **Adjusted Model: COVID-19 Concern** | | | |
| --- | --- | --- | --- | --- | --- | --- | --- | --- |
| *Predictors* | *Estimates* | *std. Error* | *Conf. Int (95%)* | *P-Value* | *Estimates* | *std. Error* | *Conf. Int (95%)* | *P-Value* |
| Intercept | 6.981 | 0.409 | 6.180 – 7.782 | **<0.001** | 1395.376 | 550.691 | 316.042 – 2474.710 | **0.011** |
| Confirmed World Cases | -0.239 | 0.030 | -0.298 – -0.181 | **<0.001** | 0.665 | 0.332 | 0.014 – 1.316 | **0.045** |
| Age |  |  |  |  | 0.011 | 0.002 | 0.007 – 0.014 | **<0.001** |
| Gender: Woman |  |  |  |  | 0.151 | 0.045 | 0.063 – 0.239 | **0.001** |
| Gender: Nonbinary |  |  |  |  | 0.271 | 0.125 | 0.026 – 0.516 | **0.030** |
| Gender: Prefer to Self Describe |  |  |  |  | 0.877 | 0.511 | -0.123 – 1.878 | 0.086 |
| Gender: Prefer not to Answer |  |  |  |  | -0.546 | 0.337 | -1.206 – 0.113 | 0.105 |
| Date |  |  |  |  | -0.076 | 0.030 | -0.136 – -0.017 | **0.012** |
| **Random Effects** | | | | | | | | |
| σ^2^ | 0.80 | | | | 0.78 | | | |
| τ_00_ | 0.03 _country_region_ | | | | 0.01 _country_region_ | | | |
| N | 24 _country_region_ | | | | 24 _country_region_ | | | |
| Observations | 1991 | | | | 1991 | | | |
| AIC | 5224.872 | | | | 5174.163 | | | |

## Table S7: Epidemiological Confirmed Country Cases Predicting COVID-19 Concern.

|  | **Unadjusted Model: COVID-19 Concern** | | | | **Adjusted Model: COVID-19 Concern** | | | |  |
| --- | --- | --- | --- | --- | --- | --- | --- | --- | --- |
| *Predictors* | *Estimates* | *std. Error* | *Conf. Int (95%)* | *P-Value* | | *Estimates* | *std. Error* | *Conf. Int (95%)* | *P-Value* |
| Intercept | 4.821 | 0.182 | 4.465 – 5.177 | **<0.001** | | 443.671 | 73.883 | 298.864 – 588.479 | **<0.001** |
| Confirmed Cases Within Country | -0.121 | 0.017 | -0.154 – -0.088 | **<0.001** | | 0.053 | 0.018 | 0.018 – 0.088 | **0.003** |
| Age |  |  |  |  | | 0.011 | 0.002 | 0.007 – 0.015 | **<0.001** |
| Gender: Woman |  |  |  |  | | 0.153 | 0.045 | 0.065 – 0.242 | **0.001** |
| Gender: Nonbinary |  |  |  |  | | 0.273 | 0.125 | 0.027 – 0.518 | **0.030** |
| Gender: Prefer to Self Describe |  |  |  |  | | 0.876 | 0.512 | -0.127 – 1.880 | 0.087 |
| Gender: Prefer not to Answer |  |  |  |  | | -0.514 | 0.337 | -1.173 – 0.146 | 0.127 |
| Date |  |  |  |  | | -0.024 | 0.004 | -0.032 – -0.016 | **<0.001** |
| **Random Effects** | | | | | | | | | |
| σ^2^ | 0.80 | | | | | 0.78 | | | |
| τ_00_ | 0.09 _country_region_ | | | | | 0.00 _country_region_ | | | |
| N | 22 _country_region_ | | | | | 22 _country_region_ | | | |
| Observations | 1986 | | | | | 1986 | | | |
| AIC | 5229.115 | | | | | 5165.397 | | | |

## Table S8: Combined Epidemiological Confirmed World and Country Cases Model Predicting COVID-19 Concern.

|  | **Epidemiological Model** | | | |
| --- | --- | --- | --- | --- |
| *Predictors* | *Estimates* | *std. Error* | *Conf. Int (95%)* | *P-Value* |
| Intercept | 1376.651 | 561.704 | 275.731 – 2477.571 | **0.014** |
| Confirmed Country Cases | 0.042 | 0.019 | 0.006 – 0.079 | **0.023** |
| Confirmed World Cases | 0.584 | 0.343 | -0.088 – 1.256 | 0.089 |
| Age | 0.011 | 0.002 | 0.007 – 0.014 | **<0.001** |
| Gender: Woman | 0.161 | 0.046 | 0.071 – 0.251 | **<0.001** |
| Gender: Nonbinary | 0.283 | 0.130 | 0.028 – 0.538 | **0.030** |
| Gender: Prefer to Self Describe | 0.891 | 0.513 | -0.115 – 1.897 | 0.082 |
| Gender: Prefer not to Answer | -0.530 | 0.338 | -1.192 – 0.132 | 0.117 |
| Date | -0.075 | 0.031 | -0.136 – -0.015 | **0.015** |
| **Random Effects** | | | | |
| σ^2^ | 0.78 | | | |
| τ_00_ _country_region_ | 0.00 | | | |
| N _country_region_ | 21 | | | |
| Observations | 1929 | | | |
| AIC | 5028.210 | | | |

## Table S9: Combined Mental Health and Epidemiological Model Predicting COVID-19 Concern.

|  | **COVID-19 Concern** | | | |
| --- | --- | --- | --- | --- |
| *Predictors* | *Estimates* | *std. Error* | *Conf. Int (95%)* | *P-Value* |
| Intercept | 820.919 | 495.823 | -150.877 – 1792.714 | 0.098 |
| GAD Score | 0.209 | 0.012 | 0.186 – 0.232 | **<0.001** |
| PHQ Score | 0.014 | 0.013 | -0.011 – 0.039 | 0.259 |
| Confirmed World Cases | 0.289 | 0.303 | -0.304 – 0.882 | 0.340 |
| Confirmed Country Cases | 0.018 | 0.017 | -0.015 – 0.050 | 0.283 |
| Age | 0.014 | 0.002 | 0.011 – 0.018 | **<0.001** |
| Gender: Woman | 0.037 | 0.041 | -0.043 – 0.117 | 0.361 |
| Gender: Nonbinary | -0.031 | 0.116 | -0.258 – 0.195 | 0.787 |
| Gender: Prefer to Self Describe | 0.787 | 0.453 | -0.100 – 1.674 | 0.082 |
| Gender: Prefer not to Answer | -0.654 | 0.298 | -1.238 – -0.070 | **0.028** |
| Date | -0.045 | 0.027 | -0.098 – 0.009 | 0.100 |
| **Random Effects** | | | | |
| σ^2^ | 0.61 | | | |
| τ_00_ _country_region_ | 0.00 | | | |
| N _country_region_ | 21 | | | |
| Observations | 1929 | | | |
| AIC | 4546.482 | | | |

**Relationship Between Financial Strain, Mental Health, and COVID-19 Concern.**

## Table S10: Loss of Employment Predicting COVID-19 Concern

|  | **Unadjusted Model: COVID-19 Concern** | | | **Adjusted Model: COVID-19 Concern** | | | | |  |
| --- | --- | --- | --- | --- | --- | --- | --- | --- | --- |
| *Predictors* | *Estimates* | *std. Error* | *Conf. Int (95%)* | | *P-Value* | *Estimates* | *std. Error* | *Conf. Int (95%)* | *P-Value* |
| Intercept | 433.798 | 49.857 | 336.080 – 531.517 | | **<0.001** | 315.096 | 52.789 | 211.630 – 418.561 | **<0.001** |
| Loss of Job | 0.158 | 0.059 | 0.042 – 0.274 | | **0.007** | 0.173 | 0.058 | 0.058 – 0.287 | **0.003** |
| Age | -0.023 | 0.003 | -0.029 – -0.018 | | **<0.001** | -0.017 | 0.003 | -0.023 – -0.011 | **<0.001** |
| Gender: Woman |  |  |  | |  | 0.011 | 0.002 | 0.008 – 0.015 | **<0.001** |
| Gender: Nonbinary |  |  |  | |  | 0.151 | 0.045 | 0.063 – 0.240 | **0.001** |
| Gender: Prefer to Self Describe |  |  |  | |  | 0.252 | 0.125 | 0.007 – 0.497 | **0.044** |
| Gender: Prefer not to Answer |  |  |  | |  | 0.903 | 0.510 | -0.097 – 1.903 | 0.077 |
| Date |  |  |  | |  | -0.530 | 0.336 | -1.188 – 0.129 | 0.115 |
| **Random Effects** | | | | | | | | | |
| σ^2^ | 0.80 | | | | | 0.78 | | | |
| τ_00_ | 0.03 _country_region_ | | | | | 0.01 _country_region_ | | | |
| N | 24 _country_region_ | | | | | 24 _country_region_ | | | |
| Observations | 1990 | | | | | 1990 | | | |
| AIC | 5213.788 | | | | | 5167.742 | | | |

## Table S11: Loss of Employment Predicting GAD-2

|  | **Unadjusted Model: GAD Scores** | | | | **Adjusted Model: GAD Scores** | | | |
| --- | --- | --- | --- | --- | --- | --- | --- | --- |
| *Predictors* | *Estimates* | *std. Error* | *Conf. Int (95%)* | *P-Value* | *Estimates* | *std. Error* | *Conf. Int (95%)* | *P-Value* |
| Intercept | 2.691 | 0.178 | 2.341 – 3.041 | **<0.001** | 86.986 | 115.124 | -138.653 – 312.626 | 0.450 |
| Loss of Job | 0.290 | 0.128 | 0.040 – 0.541 | **0.023** | 0.227 | 0.127 | -0.023 – 0.476 | 0.076 |
| Age |  |  |  |  | -0.015 | 0.004 | -0.023 – -0.007 | **<0.001** |
| Gender: Woman |  |  |  |  | 0.563 | 0.098 | 0.371 – 0.755 | **<0.001** |
| Gender: Nonbinary |  |  |  |  | 1.317 | 0.272 | 0.783 – 1.851 | **<0.001** |
| Gender: Prefer to Self Describe |  |  |  |  | 0.455 | 1.110 | -1.722 – 2.631 | 0.682 |
| Gender: Prefer not to Answer |  |  |  |  | 0.572 | 0.735 | -0.868 – 2.012 | 0.436 |
| Date |  |  |  |  | -0.005 | 0.006 | -0.017 – 0.008 | 0.465 |
| **Random Effects** | | | | | | | | |
| σ^2^ | 3.78 | | | | 3.67 | | | |
| τ_00_ | 0.26 _country_region_ | | | | 0.25 _country_region_ | | | |
| N | 24 _country_region_ | | | | 24 _country_region_ | | | |
| Observations | 1989 | | | | 1989 | | | |
| AIC | 8313.442 | | | | 8267.438 | | | |

## Table S12: Loss of Employment Predicting PHQ-2

|  | **Unadjusted Model: PHQ Scores** | | | | **Adjusted Model: PHQ Scores** | | | |
| --- | --- | --- | --- | --- | --- | --- | --- | --- |
| *Predictors* | *Estimates* | *std. Error* | *Conf. Int (95%)* | *P-Value* | *Estimates* | *std. Error* | *Conf. Int (95%)* | *P-Value* |
| Intercept | -722.015 | 98.128 | -914.342 – -529.687 | **<0.001** | -571.218 | 104.514 | -776.062 – -366.374 | **<0.001** |
| Loss of Job | 0.611 | 0.117 | 0.382 – 0.839 | **<0.001** | 0.568 | 0.116 | 0.340 – 0.795 | **<0.001** |
| Age | 0.039 | 0.005 | 0.029 – 0.050 | **<0.001** | 0.031 | 0.006 | 0.020 – 0.042 | **<0.001** |
| Gender: Woman |  |  |  |  | -0.016 | 0.004 | -0.023 – -0.009 | **<0.001** |
| Gender: Nonbinary |  |  |  |  | 0.270 | 0.089 | 0.095 – 0.445 | **0.002** |
| Gender: Prefer to Self Describe |  |  |  |  | 0.707 | 0.248 | 0.221 – 1.194 | **0.004** |
| Gender: Prefer not to Answer |  |  |  |  | 0.917 | 1.013 | -1.068 – 2.902 | 0.365 |
| Date |  |  |  |  | 1.242 | 0.666 | -0.062 – 2.547 | 0.062 |
| **Random Effects** | | | | | | | | |
| σ^2^ | 3.10 | | | | 3.06 | | | |
| τ_00_ | 0.00 _country_region_ | | | | 0.00 _country_region_ | | | |
| N | 24 _country_region_ | | | | 24 _country_region_ | | | |
| Observations | 1988 | | | | 1988 | | | |
| AIC | 7900.191 | | | | 7900.346 | | | |

# **Predictors of Degree of Adherence to Self-Quarantine Recommendations.**

## Table S13: GAD-2 Predicting Degree of Self-Quarantine

|  | **Unadjusted Model: Self-Quarantine** | | | | **Adjusted Model: Self-Quarantine** | | | |
| --- | --- | --- | --- | --- | --- | --- | --- | --- |
| *Predictors* | *Estimates* | *std. Error* | *Conf. Int (95%)* | *P-Value* | *Estimates* | *std. Error* | *Conf. Int (95%)* | *P-Value* |
| Intercept | 3.227 | 0.028 | 3.173 – 3.281 | **<0.001** | 60.918 | 37.083 | -11.764 – 133.600 | 0.100 |
| GAD Score | 0.033 | 0.007 | 0.019 – 0.047 | **<0.001** | 0.032 | 0.007 | 0.018 – 0.046 | **<0.001** |
| Age |  |  |  |  | 0.002 | 0.001 | -0.000 – 0.005 | 0.099 |
| Gender: Woman |  |  |  |  | 0.049 | 0.032 | -0.013 – 0.112 | 0.123 |
| Gender: Nonbinary |  |  |  |  | 0.136 | 0.089 | -0.038 – 0.311 | 0.126 |
| Gender: Prefer to Self Describe |  |  |  |  | 0.377 | 0.362 | -0.332 – 1.085 | 0.298 |
| Gender: Prefer not to Answer |  |  |  |  | -0.164 | 0.238 | -0.630 – 0.302 | 0.491 |
| Date |  |  |  |  | -0.003 | 0.002 | -0.007 – 0.001 | 0.119 |
| **Random Effects** | | | | | | | | |
| σ^2^ | 0.39 | | | | 0.39 | | | |
| τ_00_ | 0.00 _country_region_ | | | | 0.00 _country_region_ | | | |
| N | 24 _country_region_ | | | | 24 _country_region_ | | | |
| Observations | 1987 | | | | 1987 | | | |
| AIC | 3788.691 | | | | 3787.034 | | | |

## Table S14: PHQ-2 Predicting Degree of Self-Quarantine

|  | **Unadjusted Model: Self-Quarantine** | | | | **Adjusted Model: Self-Quarantine** | | | |
| --- | --- | --- | --- | --- | --- | --- | --- | --- |
| *Predictors* | *Estimates* | *std. Error* | *Conf. Int (95%)* | *P-Value* | *Estimates* | *std. Error* | *Conf. Int (95%)* | *P-Value* |
| Intercept | 3.245 | 0.025 | 3.197 – 3.293 | **<0.001** | 86.936 | 37.365 | 13.701 – 160.171 | **0.020** |
| PHQ Score | 0.036 | 0.008 | 0.020 – 0.051 | **<0.001** | 0.040 | 0.008 | 0.025 – 0.056 | **<0.001** |
| Age |  |  |  |  | 0.002 | 0.001 | -0.000 – 0.005 | 0.068 |
| Gender: Woman |  |  |  |  | 0.057 | 0.032 | -0.005 – 0.120 | 0.072 |
| Gender: Nonbinary |  |  |  |  | 0.150 | 0.089 | -0.024 – 0.324 | 0.090 |
| Gender: Prefer to Self Describe |  |  |  |  | 0.359 | 0.361 | -0.349 – 1.067 | 0.320 |
| Gender: Prefer not to Answer |  |  |  |  | -0.198 | 0.238 | -0.664 – 0.267 | 0.404 |
| Date |  |  |  |  | -0.005 | 0.002 | -0.009 – -0.001 | **0.025** |
| **Random Effects** | | | | | | | | |
| σ^2^ | 0.39 | | | | 0.39 | | | |
| τ_00_ | 0.00 _country_ | | | | 0.00 _country_ | | | |
| N | 24 _country_ | | | | 24 _country_ | | | |
| Observations | 1987 | | | | 1987 | | | |
| AIC | 3789.343 | | | | 3781.517 | | | |

## Table S15: Combined GAD-2 and PHQ-2 Model Predicting Degree of Self-Quarantine

|  | **Self-Quarantine** | | | |
| --- | --- | --- | --- | --- |
| *Predictors* | *Estimates* | *std. Error* | *Conf. Int (95%)* | *P-Value* |
| Intercept | 79.789 | 37.554 | 6.185 – 153.393 | **0.034** |
| PHQ Score | 0.029 | 0.010 | 0.010 – 0.049 | **0.003** |
| GAD Score | 0.016 | 0.009 | -0.002 – 0.034 | 0.077 |
| Age | 0.002 | 0.001 | -0.000 – 0.005 | 0.063 |
| Gender: Woman | 0.051 | 0.032 | -0.012 – 0.114 | 0.113 |
| Gender: Nonbinary | 0.136 | 0.089 | -0.038 – 0.310 | 0.126 |
| Gender: Prefer to Self Describe | 0.360 | 0.361 | -0.347 – 1.067 | 0.319 |
| Gender: Prefer not to Answer | -0.193 | 0.237 | -0.658 – 0.272 | 0.416 |
| Date | -0.004 | 0.002 | -0.008 – -0.000 | **0.041** |
| **Random Effects** | | | | |
| σ^2^ | 0.39 | | | |
| τ_00_ _country_ | 0.00 | | | |
| N _country_ | 24 | | | |
| Observations | 1987 | | | |
| AIC | 3780.388 | | | |

## Table S16: COVID-19 Concern Predicting Degree of Self-Quarantine

|  | **Unadjusted Model: Self-Quarantine** | | | | **Adjusted Model: Self-Quarantine** | | | |
| --- | --- | --- | --- | --- | --- | --- | --- | --- |
| *Predictors* | *Estimates* | *std. Error* | *Conf. Int (95%)* | *P-Value* | *Estimates* | *std. Error* | *Conf. Int (95%)* | *P-Value* |
| Intercept | 2.783 | 0.061 | 2.663 – 2.902 | **<0.001** | 23.613 | 36.817 | -48.546 – 95.772 | 0.521 |
| COVID-19 Concern | 0.141 | 0.015 | 0.112 – 0.171 | **<0.001** | 0.136 | 0.016 | 0.106 – 0.167 | **<0.001** |
| Gender: Woman |  |  |  |  | 0.047 | 0.031 | -0.015 – 0.109 | 0.134 |
| Gender: Nonbinary |  |  |  |  | 0.143 | 0.087 | -0.028 – 0.315 | 0.101 |
| Gender: Prefer to Self Describe |  |  |  |  | 0.270 | 0.357 | -0.429 – 0.970 | 0.449 |
| Gender: Prefer not to Answer |  |  |  |  | -0.074 | 0.235 | -0.534 – 0.386 | 0.752 |
| Age |  |  |  |  | 0.000 | 0.001 | -0.002 – 0.003 | 0.879 |
| Date |  |  |  |  | -0.001 | 0.002 | -0.005 – 0.003 | 0.571 |
| **Random Effects** | | | | | | | | |
| σ^2^ | 0.38 | | | | 0.38 | | | |
| τ_00_ | 0.00 _country_region_ | | | | 0.00 _country_region_ | | | |
| N | 24 _country_region_ | | | | 24 _country_region_ | | | |
| Observations | 1990 | | | | 1990 | | | |
| AIC | 3729.120 | | | | 3736.136 | | | |

## Table S17: Epidemiological Total World Cases Model Predicting Degree of Self-Quarantine

|  | **Unadjusted Model: Self Quarantite** | | | | **Adjusted Model: Self-Quarantine** | | | |
| --- | --- | --- | --- | --- | --- | --- | --- | --- |
| *Predictors* | *Estimates* | *std. Error* | *Conf. Int (95%)* | *P-Value* | *Estimates* | *std. Error* | *Conf. Int (95%)* | *P-Value* |
| Intercept | 3.934 | 0.285 | 3.376 – 4.492 | **<0.001** | 626.233 | 391.429 | -140.954 – 1393.420 | 0.110 |
| Confirmed World Cases | -0.044 | 0.021 | -0.085 – -0.003 | **0.036** | 0.341 | 0.236 | -0.121 – 0.804 | 0.148 |
| Age |  |  |  |  | 0.002 | 0.001 | -0.001 – 0.004 | 0.250 |
| Gender: Woman |  |  |  |  | 0.068 | 0.032 | 0.006 – 0.131 | **0.032** |
| Gender: Nonbinary |  |  |  |  | 0.183 | 0.089 | 0.009 – 0.357 | **0.040** |
| Gender: Prefer to Self Describe |  |  |  |  | 0.394 | 0.363 | -0.318 – 1.105 | 0.278 |
| Gender: Prefer not to Answer |  |  |  |  | -0.159 | 0.239 | -0.628 – 0.309 | 0.504 |
| Date |  |  |  |  | -0.034 | 0.022 | -0.076 – 0.008 | 0.112 |
| **Random Effects** | | | | | | | | |
| σ^2^ | 0.40 | | | | 0.39 | | | |
| τ_00_ | 0.00 _country_region_ | | | | 0.00 _country_region_ | | | |
| N | 24 _country_region_ | | | | 24 _country_region_ | | | |
| Observations | 1990 | | | | 1990 | | | |
| AIC | 3810.169 | | | | 3808.863 | | | |

## Table S18: Epidemiological Total Country Cases Model Predicting Degree of Self-Quarantine

|  | **Unadjusted Model: Self-Quarantine** | | | | **Adjusted Model: Self-Quarantine** | | | |
| --- | --- | --- | --- | --- | --- | --- | --- | --- |
| *Predictors* | *Estimates* | *std. Error* | *Conf. Int (95%)* | *P-Value* | *Estimates* | *std. Error* | *Conf. Int (95%)* | *P-Value* |
| Intercept | 3.428 | 0.097 | 3.238 – 3.618 | **<0.001** | 78.597 | 50.007 | -19.414 – 176.609 | 0.116 |
| Confirmed Cases Within Country | -0.008 | 0.008 | -0.024 – 0.009 | 0.347 | 0.005 | 0.011 | -0.017 – 0.028 | 0.633 |
| Age |  |  |  |  | 0.002 | 0.001 | -0.001 – 0.004 | 0.202 |
| Gender: Woman |  |  |  |  | 0.068 | 0.032 | 0.006 – 0.131 | **0.033** |
| Gender: Nonbinary |  |  |  |  | 0.181 | 0.089 | 0.006 – 0.355 | **0.042** |
| Gender: Prefer to Self Describe |  |  |  |  | 0.390 | 0.363 | -0.322 – 1.103 | 0.283 |
| Gender: Prefer not to Answer |  |  |  |  | -0.145 | 0.239 | -0.613 – 0.323 | 0.544 |
| Date |  |  |  |  | -0.004 | 0.003 | -0.009 – 0.001 | 0.132 |
| **Random Effects** | | | | | | | | |
| σ^2^ | 0.40 | | | | 0.39 | | | |
| τ_00_ | 0.00 _country_region_ | | | | 0.00 _country_region_ | | | |
| N | 24 _country_region_ | | | | 24 _country_region_ | | | |
| Observations | 1990 | | | | 1990 | | | |
| AIC | 3813.680 | | | | 3810.726 | | | |

## Table S19: Epidemiological Total World and Country Cases Combined Model Predicting Degree of Self-Quarantine

|  | **Self-Quarantine** | | | |
| --- | --- | --- | --- | --- |
| *Predictors* | *Estimates* | *std. Error* | *Conf. Int (95%)* | *P-Value* |
| Intercept | 595.274 | 393.455 | -175.884 – 1366.432 | 0.130 |
| Confirmed Country Cases | 0.003 | 0.012 | -0.020 – 0.026 | 0.806 |
| Confirmed World Cases | 0.318 | 0.240 | -0.152 – 0.788 | 0.185 |
| Gender: Woman | 0.067 | 0.032 | 0.004 – 0.130 | **0.036** |
| Gender: Nonbinary | 0.182 | 0.089 | 0.007 – 0.356 | **0.041** |
| Gender: Prefer to Self Describe | 0.391 | 0.363 | -0.321 – 1.103 | 0.282 |
| Gender: Prefer not to Answer | -0.158 | 0.239 | -0.626 – 0.311 | 0.509 |
| Age | 0.002 | 0.001 | -0.001 – 0.004 | 0.258 |
| Date | -0.032 | 0.022 | -0.075 – 0.010 | 0.133 |
| **Random Effects** | | | | |
| σ^2^ | 0.39 | | | |
| τ_00_ _country_region_ | 0.00 | | | |
| N _country_region_ | 24 | | | |
| Observations | 1987 | | | |
| AIC | 3806.823 | | | |

## Table S20: Mental Health, COVID-19 Concern, and Epidemiological Confirmed World and Country Cases Combined Model Predicting Degree of Self-Quarantine

|  | **Complete Model** | | | |
| --- | --- | --- | --- | --- |
| *Predictors* | *Estimates* | *std. Error* | *Conf. Int (95%)* | *P-Value* |
| Intercept | 409.471 | 386.170 | -347.408 – 1166.350 | 0.289 |
| GAD Score | -0.012 | 0.010 | -0.031 – 0.007 | 0.219 |
| PHQ Score | 0.028 | 0.010 | 0.008 – 0.047 | **0.005** |
| Confirmed Country Cases | -0.001 | 0.011 | -0.024 – 0.021 | 0.913 |
| Confirmed World Cases | 0.225 | 0.235 | -0.236 – 0.686 | 0.338 |
| COVID-19 Concern | 0.132 | 0.018 | 0.097 – 0.166 | **<0.001** |
| Gender: Woman | 0.046 | 0.032 | -0.016 – 0.108 | 0.144 |
| Gender: Nonbinary | 0.140 | 0.088 | -0.031 – 0.312 | 0.109 |
| Gender: Prefer to Self Describe | 0.257 | 0.356 | -0.441 – 0.956 | 0.470 |
| Gender: Prefer not to Answer | -0.114 | 0.235 | -0.574 – 0.346 | 0.627 |
| Age | 0.000 | 0.001 | -0.002 – 0.003 | 0.760 |
| Date | -0.022 | 0.021 | -0.064 – 0.019 | 0.293 |
| **Random Effects** | | | | |
| σ^2^ | 0.38 | | | |
| τ_00_ _country_region_ | 0.00 | | | |
| N _country_region_ | 24 | | | |
| Observations | 1987 | | | |
| AIC | 3730.853 | | | |

# Sample Descriptives


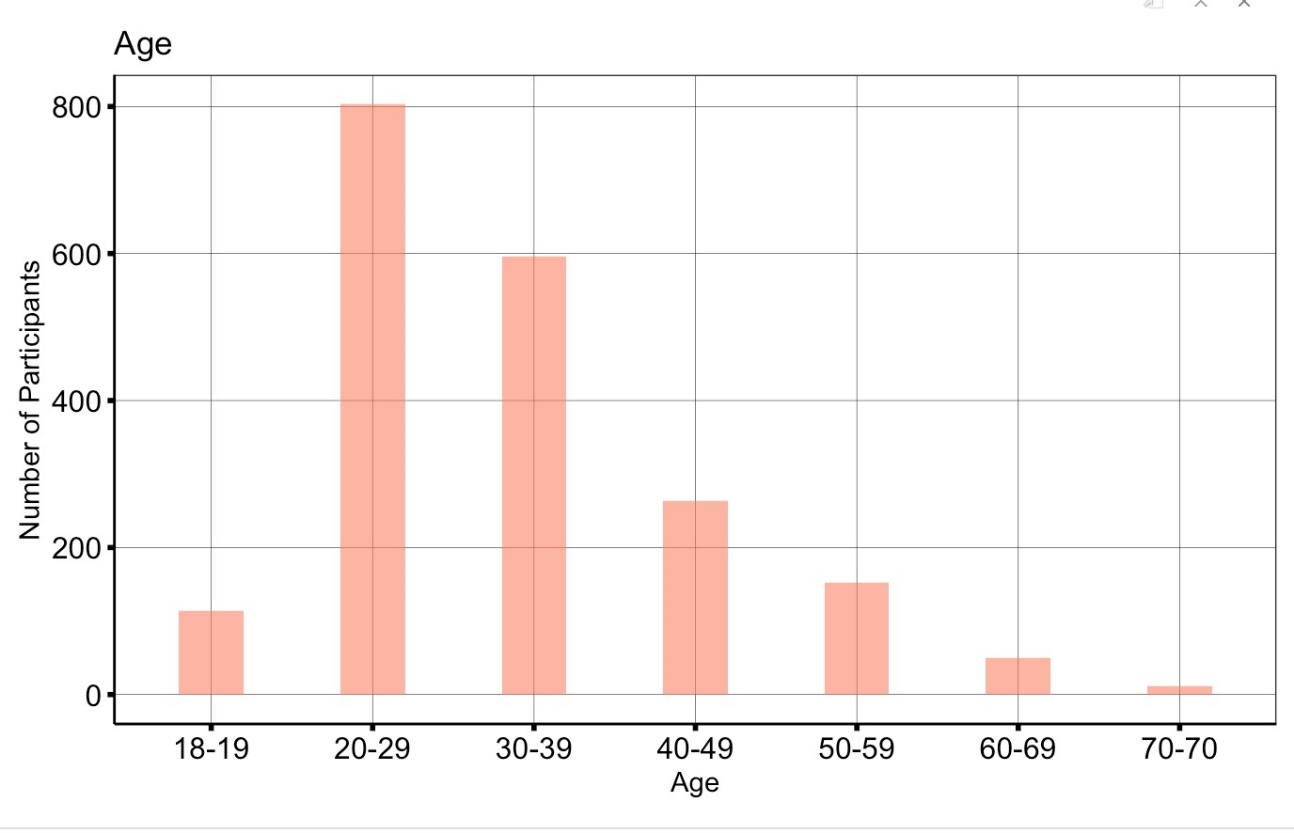


## Figure S1. Age Distribution

## Table S21. Participants by Country

| Country | N | Percent |
| --- | --- | --- |
| United States | 1683 | 81.50 |
| Canada | 137 | 6.63 |
| United Kingdom | 94 | 4.55 |
| Germany | 29 | 1.40 |
| Netherlands | 20 | 0.97 |
| France | 16 | 0.77 |
| Ireland | 16 | 0.77 |
| Sweden | 10 | 0.48 |
| Romania | 8 | 0.39 |
| Croatia | 6 | 0.29 |
| Italy | 6 | 0.29 |
| Spain | 6 | 0.29 |
| Austria | 5 | 0.24 |
| Denmark | 4 | 0.19 |
| Greece | 4 | 0.19 |
| Russian Federation | 4 | 0.19 |
| Belgium | 3 | 0.15 |
| Finland | 3 | 0.15 |
| Switzerland | 3 | 0.15 |
| Albania | 2 | 0.10 |
| Bulgaria | 2 | 0.10 |
| Serbia | 2 | 0.10 |
| Czech Republic | 1 | 0.05 |
| Luxembourg | 1 | 0.05 |
